# Placenta Praevia*Read before the Houston County Medical Society, July 15, 1909.

**Published:** 1909-09

**Authors:** R. W. Skipper

**Affiliations:** Lovelady, Texas


For Texas Medical Journal.

### Placenta Praevia.*

BY DR. R. W. SKIPPER, LOVELADY, TEXAS.

### DEFINITION.

Implantation and development of the placenta in the zone of dilatation of the uterus.

Hemorrhage from this cause is called unavoidable.

### FREQUENCY.

Placenta praevia occurs from one to four times in one thousand cases of gestation.

---

*Read before the Houston County Medical Society, July 15, 1909.

It will depend upon the practice of those who get up statistics as to frequency.

When one is engaged in a private practice, with no consultations, it is not likely that the ratio would exceed one in one thousand, but when one is often called in consultation, he may see as many as four cases in a thousand.

### VARIETIES.

Placenta praevia is divided into three classes or varieties.

First. Placenta praevia centralis, in which the organ completely covers the os uteri, when it is fully dilated.

This form is rarer than the other two, but the most dangerous of all.

Second. This variety is that in which the placenta is situated more on one side than the other, and does not quite cover the os when it is fully dilated, and is known as placenta praevia partialis.

This form is more common than the first, but less so than the third, and occupies a middle ground as to fatalities.

The placenta in this class of cases does not cover the os completely when it is fully dilated.

Third. This is the most common variety, and is called placenta praevia marginalis or lateralis.

In it the afterbirth does not extend over the internal os, but down to it. There is less danger to life here, for the uterus may dilate its lower segment with very little hemorrhage occurring because the placenta is situated above the dilating zone or partially so. It is quite apparent that this is the least dangerous to life of all varieties of this peculiar affection.

A good classification is, into complete and incomplete placenta praevia, the central type belonging to the first, or centralis, while all other varieties are comprised in the second.

### ETIOLOGY.

Multiparity is one of the most frequent causes. Endometritis, due to any cause, predisposes to placenta praevia, for with it we have a patulous os. It is said to be the cause of very many cases.

Abortions, for the same reason, will cause the trouble. Tumors may be assigned as a cause and an abnormally low insertion of the Fallopian tubes. Uterine malformations may give rise to it.

### THE CAUSE OF HEMORRHAGE.

Separation of the afterbirth from its uterine attachment is the most common cause, and the amount of blood lost will correspond to amount of separation that has taken place and the danger to life, to the amount of blood that is lost.

When the implantation is over the os there will be a great deal of blood lost, for then it covers a larger portion of that part of the uterus which dilates during labor.

The upper portion of the womb is possessed of properties of contractility and of retractility, but not of dilatability, as is the lower segment, hence the cause of antepartum hemorrhage in placenta praevia.

Edgar tells us, too, that when the placenta is implanted upon the lower segment, damage is much more likely to result from shock, jars, etc.

### PATHOLOGY.

When the placenta is centrally implanted, it is ill-shaped and much larger than when normally situated, consequently it has more surface to separate and there is more bleeding than if it were smaller

The insertion of the cord is apt to be near one edge and this gives rise to prolapse of the funis in many cases.

### SYMPTOMS.

The leading symptom is hemorrhage, and any loss of blood during the last three months of gestation should be looked upon with suspicion. Dr. Joseph B. De Lee, in the fifth volume of practical medicine series, 1907, says that 84 per cent of our cases will have hemorrhage during the last trimester, and that any painless, causeless uterine hemorrhage during the last three months of pregnancy should be taken as almost pathognomonic. The same author says that the first hemorrhage will usually be small, but followed by others that will be more or less severe, and that there may be a flooding that is so severe as to cause the death of the woman in a few minutes. If the life of the patient is not sacrificed at once, she will have a severe form of anaemia that may cause her death later on. Profuse hemorrhages point to placenta praevia totalis. They may occur earlier than in partial presentations of the placenta. Shall hemorrhages often repeated during the last trimester point to a praevia partialis.

Of course the anaemia that follows hemorrhages is only a secondary symptom and may always be expected to follow any great loss of blood.

### DIAGNOSIS.

Dr. De Lee says that the only positive sign of placenta praevia is the palpation of placental tissue through the dilated os, and that if the cervix is closed we can only suspect the condition more or less strongly. He gives as auxiliary data, bogginess in the vaginal vaults, the sensation of a flat sponge between the finger and the foetal head, pulsating arteries in the fornices, a low so-called (but erroneously) placental souffle, usually succulence and vascularity of the parts. He charges us to be careful in our manipulation, so as to avoid further separation of the placenta or tearing of the organ, which might give rise to a fatal hemorrhage, and directs us to have all preparations made to arrest hemorrhage, before making digital examination.

### PROGNOSIS.

This will depend largely upon the character of the individual case. If we have a complete presentation, then we may expect very grave results to follow upon the advent of uterine contractions and consequent separation of placental tissue and the unavoidable hemorrhage that follows. Much will depend upon the skill of the accoucheur. The mother may lose her life from hemorrhage, dying while he is making efforts to deliver her, or soon afterward, either from anaemia or sepsis. The child will be in danger in proportion to the disturbance of its circulation and nutrition.

Our prognosis will depend greatly upon the time the case is first seen, and when the diagnosis is made. If the condition is recognized early, that is before there is much blood lost, the chances for both mother and child are much better than if labor has gone on for some time and a severe hemorrhage has occurred.

In complete placenta praevia we have a grave condition, and the obstetrician who has such a case present itself to him will feel need for counsel and will appreciate your efforts to come to his assistance as soon as you can do so.

Dr. De Lee had forty-seven cases with but two deaths. He does not tell us what kind of cases his were, whether complete or incomplete, or whether seen early or late. He does not tell us the time of gestation at which labor occurred, or whether it was spontaneous or induced. I am sorry that he did not give us such data, for his is the latest that we have upon this condition. He says a woman with placenta praevia should not die except in very rare instances, such as air embolism, hemorrhagic diathesis or spontaneous rupture of the uterus.

TREATMENT.

Almost every case of placenta praevia requires immediate interference. Soon as a diagnosis is made labor is to be inaugurated. This set rule might not apply to cases in which an early diagnosis has been made. Both De Lee and Edgar say that we may before the beginning of the seventh month treat tentatively. The consensus of opinion is that the woman be made to rest. She must avoid all exercise that would bring on or foster muscular contractions, must not strain at stool, coitus must be inhibited. Opiates must be given for pain indicating uterine contractions. When hemorrhage is present the foot of the bed must be raised and cold applications made to the pelvis. One who can deal with a hemorrhage should always be by the woman. The risk to life is too great for us to ignore this rule, although its execution may not always appear feasible. A well applied tampon should be introduced when dilatation is not sufficiently advanced for us to deal with the case in a more pronounced way. Burger and Graf condemn the use of a tampon at any time because of the possibility of concealed hemorrhage taking place, yet our latest and best American authors advise their use in all cases. Sterile gauze should be used and must be packed tightly into the vagina, to prevent as far as possible the bleeding until the cervix has been obliterated, when measures can be used for rapid dilatation.

When dilatation has become sufficient to do so, one of the many bags that are made for the purpose may be introduced and filled with water and traction made upon its stem at any time when bleeding becomes great enough to demand it. All instruments that are used must be aseptic. To make them so there is nothing superior to a .5 to 1 per cent lysol solution.

When the os is sufficiently patulous to permit the introduction of a finger or a small Goodell dilator rapid dilatation should be begun and carried on as rapidly as is compatible with safety. Separating the placenta from its uterine attachment must be avoided as far as is possible, for this constitutes the chief source of danger. As soon as the os is dilated enough to admit two or three fingers a Braxton Hicks or bipolar version should be undertaken. When a foot can be gotten it should be brought down and delivered until the half breach presses upon the afterbirth, which gives us, according to good authorities, almost complete control over the hemorrhage. We are warned to be contented with the advantage thus given us, yet I fear that in our great anxiety to terminate the labor we will use too much force upon a partially dilated os, that may tear easily. The writer is sure that he has torn a cervix by using force before the maternal parts were prepared for complete delivery. This subjects our patient to added danger, for the bleeding from a small tear in the vascular uterus may be sufficient to cause her death. If she does not succumb to the loss of blood, she may die afterward from septicaemia, resulting from the nidus formed by the laceration. The ovoid buttock of the foetus pressing upon the placenta and upon its bleeding site is apt to control the hemorrhage.

If the Braxton Hicks version can not be done quickly, then we should lose no time in passing the hand into the uterus and performing an ordinary podalic version, bringing down one or both feet, as in our judgment is best. Remember, that our object is to save blood and that our efforts must be to bring the child's hips down and use them as a tampon from above downward. We are told that we must separate the placenta from its uterine attachments as little as is possible during our manipulation. The reason for this is obvious, for the bleeding will be in proportion to the uterine surface that is left bare by placental detachment.

For the induction of labor we are advised to rupture the membranes, but in cases of placenta praevia centralis, we will probably not be able to do so, for we can not reach them. I believe a good tampon should be used in such cases, for it is an excitor of uterine contractions. This will apply to cases with or without hemorrhage.

We are advised to go through the placenta in some cases, when its margin can not be reached without too much separation. This, I believe, is good advice, but we must not undertake to do it until we have a sufficient amount of dilatation to do a version at once and control the bleeding by pressure.

There is still another method of delivery spoken of, but it has fallen into disrepute, and rightly so, I should think. I refer to what is called "accouchment force," which consists of rapid dilatation either with instruments or the fingers, until the parturient canal has been sufficiently widened to permit of a version and of forcible delivery after the turning has been done. This was the rule not many decades ago, but we would use less force and more conservatism at the present time.

As stated above, we are apt to use too much force in our anxious state of mind.

### THIRD STAGE.

When the foetus has been delivered it should be given to an assistant whether living or dead, and all our attention given to the woman. The placenta should be delivered as quickly as possible. The patient is already under an anesthetic, or partially so, and manual delivery can be easily accomplished, unless the afterbirth is adherent, and if so the hand should be introduced and the organ peeled from its site. Postpartum hemorrhage may be severe even after complete delivery and will be a source of great danger. It is best controlled by manual measures. Grasp the uterus at its fundus and bend it down over the os pubis and at the same time grasp its lower segment with the other hand in the vagina, and by bringing the hands as near together as possible, put it into a small mass. Sterile gauze well packed into the womb will control bleeding when the organ is emptied and should be tried. The cavity of the uterus must be completely filled, or there may be a concealed hemorrhage, which might destroy the life of our patient, while we were feeling that she was secure. Ergot should be used, the best preparation being given by the mouth, or an aseptic ergot given by the hypodermic method. If strychnine is added it will do great good. The dose must be large. When much blood has been lost we should resort to a free infusion of normal salt solutions. Hypodermoclysis is best, using the solution under the mammary gland or in the flank. Ether may be given hypodermically and general measures used to resuscitate the exsanguinated woman.

---

LOCAL applications of tincture of iodine is one of the most satisfactory treatments for small chronic ulcers.—*American Journal of Surgery.*

---

THE meadowlark will chirp and sing, and the bumblebee will bumble, the colt will do a Highland fling, and the tumblebug will tumble, the calf will buck and jump for joy of simply being loose, the droll grasshoppers sit around and spit tobacco juice, the luckless tramp resume his march and the bulldog chase and bite him, and the horsefly irritate the mule, and so on,—*ad infinitum.—Ganderbone for June.*

